# Analysis of heat shock protein 70 gene polymorphisms Mexican patients with idiopathic pulmonary fibrosis

**DOI:** 10.1186/s12890-015-0127-7

**Published:** 2015-10-24

**Authors:** Arnoldo Aquino-Gálvez, Georgina González-Ávila, Martha Pérez-Rodríguez, Oswaldo Partida-Rodríguez, Miriam Nieves-Ramírez, Inocencio Piña-Ramírez, Gustavo Ramírez-Martínez, Manuel Castillejos-López, Marco Checa, Victor Ruiz, Francisco Urrea, Bettina Sommer, Joaquin Zúñiga, Moisés Selman

**Affiliations:** Instituto Nacional de Enfermedades Respiratorias “Ismael Cosío Villegas” Tlalpan 4502, Col. Sección XVI, 14080 Mexico, México; Hospital General de México, Universidad Nacional Autónoma de México, Laboratorio de Inmunología, Mexico, Mexico; Unidad de Investigación Médica en Inmunología, CMN S-XXI Instituto Mexicano del Seguro Social, Mexico, Mexico

**Keywords:** Idiopathic pulmonary fibrosis, Single nucleotide polymorphisms, Heat shock proteins of 70 kDa (HSP70), MHC polymorphisms

## Abstract

**Background:**

Idiopathic pulmonary fibrosis (IPF) is a fatal lung disease of unknown etiology. Genetic variation within different major histocompatibility complex (MHC) loci contributes to the susceptibility to IPF. The effect of 70 kDa heat shock proteins (*HSP70*) gene polymorphisms in the susceptibility to IPF is unknown. The aim of this study was to explore the association between HSP70 polymorphisms and IPF susceptibility in the Mexican population.

**Methods:**

Four HSP70 single nucleotide polymorphisms (SNPs) were evaluated using real time PCR assays in 168 IPF patients and 205 controls: +2763 C>T of *HSPA1L* (rs2075800), +2437 of *HSP HSPA1L* A>G (rs2227956), +190 of *HSPA1A* G>C (rs1043618) and +1267 of *HSPA1B* G>A (rs1061581).

**Results:**

The analysis of the recessive model revealed a significant decrease in the frequency of the genotype HSPA1B AA (rs1061581) in IPF patients (OR = 0.27, 95 % CI = 0.13–0.57, Pc = 0.0003) when compared to controls. Using a multivariate logistic regression analysis in a codominant model the *HSPA1B* (rs1061581) GA and AA genotypes were associated with a lower risk of IPF compared with GG (OR = 0.22, 95 % CI = 0.07–0.65; *p* = 0.006 and OR = 0.17, 95 % CI = 0.07–0.41; *p* = <0.001). Similarly, *HSPA1L* (rs2227956) AG genotype (OR = 0.34, 95 % CI = 0.12–0.99; *p* = 0.04) and the dominant model AG + GG genotypes were also associated with a lower risk of IPF (OR = 0.24, 95 % CI = 0.08–0.67; *p* = 0.007). In contrast, the *HSPA1L* (rs2075800) TT genotype was associated with susceptibility to IPF (OR = 2.52, 95 % CI = 1.32–4.81; *p* = 0.005).

**Conclusion:**

Our findings indicate that HSPA1B (rs1061581), HSPA1L (rs2227956) and *HSPA1* (rs1043618) polymorphisms are associated with a decreased risk of IPF.

**Electronic supplementary material:**

The online version of this article (doi:10.1186/s12890-015-0127-7) contains supplementary material, which is available to authorized users.

## Background

Idiopathic pulmonary fibrosis (IPF) is a progressive and usually lethal disease of unknown etiology characterized by alveolar epithelial cell activation, fibroblast/myofibroblasts proliferation and activation and exaggerated accumulation of extracellular matrix (ECM) in lung parenchyma [1]. A wide variety of genetic risk factors likely involved in susceptibility to develop IPF have been described including common variants in *MUC5B* [2], *TERT*, a component of telomerase [2, 3], Toll-interacting protein (*TOLLIP*) [4], surfactant protein A and B [5] and polymorphisms within the major histocompatibility complex (MHC) [6]. In this context, we have reported that MHC class I chain-related gene A (*MICA*) polymorphisms might also contribute to IPF susceptibility in Mexicans [7]. These findings and the multifactorial nature of IPF suggest that there may be other unidentified genetic factors within the MHC region involved in IPF susceptibility. *HSP70* genes are coded within MHC class III region and their products are involved in the binding and stabilization of nascent peptides for their correct folding to achieve appropriate conformations and in the removal of misfolded and denaturized proteins [8–10]. Emerging evidence indicates that endoplasmic reticulum (ER) stress and activation of the unfolded protein response (UPR) may play a role in the pathogenesis of IPF [11]. Variations in the sequence of *HSP70* genes appear to affect the expression or function of HSP70 proteins resulting in altered stress tolerance mechanisms and contributing to the susceptibility to different pathological conditions [12]. *HSP70* gene variations are also associated with alterations in oxidative stress [13–22]. There are three major genes of the family of human HSP70 within the MHC class III region; these genes are *HSP70*-1 (*HSPA1A*, OMIM: 140550), *HSP70*-2 (*HSPA1B*; OMIM: 603012) and *HSP70*-HOM (*HSP70*A1L; OMIM: 140559) [23]. The products of the first two genes encode a similar heat-inducible Hsp70 protein that differ only in two amino acids; whereas *HSP70*-HOM encodes a non–heat-inducible protein that shares high homology with the protein products of HSP70-1 and HSP70-2 [8–10, 24]. Some report functional consequences of the *HSP70* SNPs, for example in the +190 C allele of *HSPA1A*, located in the 5′ UTR region, provokes a reduction in the promoter activity and HSP70 protein expression than +190 G allele [25]. The polymorphisms *HSPA1B*-179 C>T and *HSPA1B* 1267 A>G have been also associated with differential production of HSPA1A and HSPA1B mRNA [26]. Also, the *HSP70-HOM* +2437 A>G (Met493Thr) polymorphism in the peptide-binding domain appears to affect the substrate specificity and chaperone activity of this protein [12, 27].

The aim of the present study was to examine the possible association of *HSP70* gene polymorphisms with the susceptibility to IPF in the Mexican population.

## Methods

### Patients

One hundred sixty eight Mexican patients with diagnosis of IPF were included in this study (103 males, 65 females, 64.5 ± 11.0 years old). Patients were recruited from the Interstitial Lung Diseases Clinic of the Instituto Nacional de Enfermedades Respiratorias “Ismael Cosio Villegas”. Diagnosis of IPF was made with the currently accepted international criteria [28].

Patients with known causes of interstitial lung disease (i.e., collagen vascular disease, drug toxicity, environmental exposure) were excluded.

As controls, we included 205 unrelated healthy volunteers (36 males, 169 females), with an average age of 47 ± 5.4 years. Control subjects included non-smokers and smokers with normal lung function.

Patients and controls were individuals with the same ethnic origin and with at least two generations born in Mexico.

Written informed consent letter was obtained from all patients and controls. The protocol was reviewed and approved by the Scientific and Ethics Institutional Review Board of the Instituto Nacional de Enfermedades Respiratorias “Ismael Cosio Villegas”.

### DNA isolation

Venous blood samples were collected in 5-ml EDTA coated tubes from IPF patients and controls and DNA was isolated using a BDtract genomic DNA isolation kit (Maxim Biotech, San Francisco CA).

### TaqMan 5' genotyping allelic discrimination assay

Variations of *HSPA1L* +2763 C>T (rs2075800), +2437 of *HSPA1L* A>G (rs2227956), + 190 of *HSPA1A* G>C (rs1043618) and +1267 of *HSPA1B*G>A (rs1061581) were genotyped by predesigned 5’ nuclease SNP genotyping assays in accordance with the manufacturer protocol (Applied Biosystems Foster City, CA). The selection of these SNPs was based on the availability of previous studies regarding gene and allele frequencies in different ethnic groups. The reagents included primers and allele-specific probes 5’- labeled with VIC or FAM fluorochromes to detect the alleles of *HSPA1*. Each reaction contained 10 ng of genomic DNA, TaqMan Universal PCR Master Mix (PE Applied Biosystems), 900 nM primers, and 50 nM probes in 25 μl. The analyses were performed using an ABI Prism Step One Real time PCR System (Applied Biosystems Foster City, CA). Thermal cycling conditions were 2 min at 50 °C, 10 min at 95 °C and 40 cycles each of 95 °C for 15 s and 50 °C for 1 min.

### Statistical analysis

Hardy–Weinberg equilibrium (HWE) was tested for all genotypic combinations of each variant using the Haploview software (Version 4.2) [29]. The allelic and genotypic frequencies were determined by direct counting in patients and controls. Differences in allele, genotype and haplotype frequencies were evaluated by the Pearson Chi-square test that combined the 2 × 2 contingency tables in IPF patients and control group using the EPIINFO statistical program (Version 6.04b). Corrected P (Pc) values, odds ratio (OR) with 95 % confidence interval (CI) were also estimated using EPIINFO. Statistical significance of associations with minor allele positivity (dominant model) or minor allele homozygosis (recessive model) was assessed by OR and their 95 % CI were obtained. In these models, the wild-type homozygous group was the reference group for comparisons [21].

For genotypes the value "p" of hypothesis testing between 4 polymorphisms under the 3 different models of inheritance and the presence or absence of FPI was adjusted. The test used was the Bonferroni correction and the calculation was carried out as follows: the level alpha/number of comparisons, the number of comparisons was determined by the number of SNPs (four) multiplied by the number of models of inheritance (three) between the alpha value that was 0.05 and the result was 0.004. This indicates that the values of p <0.004 can be considered statistically significant. For the alleles, the number of comparisons was determined by the number of alleles (four), between the alpha value (0.05); the result was 0.0125 and this indicates that any values of p <0.0125 can be considered statistically significant. We decided to use genotypes as the primary comparison due to the interest of establishing the possible association from models of Mendelian inheritance: dominance, co-dominant and recessive; furthermore, this approach gives more comprehensive information these polymorphisms’ role in this disease. The hypothesis was built under the dominant pattern of inheritance; the calculation power for this study was 0.885, this was calculated in the Power and Sample Size software [30]. To adjust the estimates and determine if these protection associations are independent, we performed a multivariate logistic regression analysis for four SNPs of *HSP70* and their association with the IPF for three models of genetic inheritance.

## Results

The allele and frequencies of *HSP70* gene SNPs are shown in Table 1. A decreased frequency of the G allele of the SNP rs2227956 of *HSPA1L* gene was observed in IPF patients when compared to controls (OR = 0.27, 95 % CI = 0.10–0.75, Pc = 0.01). The genotype frequencies and the ORs for the codominant, dominant and recessive models are also shown in Table 1. A significant decrease in the frequency of the heterozygous AG genotype of the SNP rs2227956 of *HSPA1L* gene (OR = 0.26, 95 % CI = 0.09–0.72, Pc = 0.01,) and in the frequency of the homozygous AA genotype of the SNP rs1061581 of the *HSPA1B* gene was also found in the IPF group (OR = 0.30, 95 % CI = 0.13–0.57, Pc = 0.001) when compared to the control group. These associations were observed in co-dominant and dominant models for genotype AG of *HSPA1L* (rs2227956) and co-dominant and recessive model for genotype AA of *HSPA1B* (rs1061581) (Table 1). To adjust the estimates and determine if these genetic associations with IPF are independent, we performed a multivariate logistic regression analysis of the four SNPs of *HSP70* and their association with the disease for three models of genetic inheritance (Table 2). The pattern observed in codominant *HSPA1B* (rs1061581) GA genotype was associated with a lower risk, compared with GG (OR = 0.22, 95 % CI = 0.07–0.65; *p* = 0.006), whereas the comparison AA vs GG displayed a stronger protective association with IPF (OR = 0.17, 95 % CI = 0.07–0.41; *p* = <0.001). In contrast, *HSPA1L* (rs2075800) TT genotype confers an increased risk for the development of IPF (OR = 2.52, 95 % CI = 1.32–4.81; *p* = 0.005). In the dominant model analysis we found that the genotypes AG + GG of HSPA1 (rs2227956) (OR = 0.24, 95 % CI = 0.089–0.679; *p* = 0.007) as well as the combination of genotypes GC + CC (OR = 0.639, 95 % CI = 0.41–0.97; *p* = 0.039) of HSPA1 (rs1043618) confer protection to IPF. In the recessive model analysis we found that the AA genotype of *HSPA1B* (rs1061581) seems equally protective (OR = 0.68, 95 % CI = 0.07–0.03; *p* = <0.001), whereas the TT genotype confers a risk for IPF development (OR = 2.70, 95 % CI = 1.46–4.98; *p* = 0.001).

**Table 1:** *HSPA1L*, *HSPA1A* and *HSPA1B* genotype and allele frequencies in IPF patients and healthy controls

		IPF *n* = 168		Control *n* = 205				
Models	Allele/Genotypes	n	f	n	f	OR	Pc	95 % C.I.
	*HSPA1L* (C/T rs2075800)							
	*C*	181	0.79	230	0.56	1		
	*T*	155	0.21	180	0.44	1.09	0.54	0.81–1.46
C	*CC*	53	0.31	59	0.28	1	–	–
	*CT*	75	0.44	112	0.54	0.74		–
	*TT*	40	0.23	34	0.16	1.30	0.10	0.72–2.35
D	*CC*	53	0.31	59	0.28	1	–	–
	*CT + TT*	115	0.68	146	0.71	0.87	0.56	0.56–1.36
R	*CC + CT*	128	0.76	171	0.83	1	–	–
	*TT*	40	0.23	34	0.16	1.57	0.08	0.94–2.62
	*HSPA1L* (A/G rs2227956)							
	*A*	331	0.99	389	0.95	1	–	–
	*G*	5	0.01	21	0.05	0.27	0.01	0.10–0.75
C	*AA*	163	0.97	184	0.89	1	–	–
	*AG*	5	0.02	21	0.10	0.26	0.01	0.09–0.72
	*GG*	0	0.00	0	0.00	–	–	–
D	*AA*	163	0.97	184	0.89	1	–	–
	*AG + GG*	5	0.02	21	0.10	0.26	0.01	0.09–0.72
R	*AA + AG*	168	1.00	205	1.00	1	–	–
	*GG*	0	0.00	0	0.00	–	–	–
	*HSPA1A* (G/C rs1043618)							
	*G*	261	0.77	295	0.72	1	–	–
	*C*	75	0.22	115	0.28	0.73	0.07	0.52–1.03
C	*GG*	100	0.59	102	0.49	1	–	–
	*GC*	61	0.36	91	0.44	0.68		–
	*CC*	7	0.04	12	0.05	0.59	0.16	0.22–1.57
D	*GG*	100	0.59	102	0.49	1	–	–
	*GC + CC*	68	0.40	103	0.50	0.68	0.07	0.45–1.03
R	*GG + GC*	161	0.95	193	0.94	1	–	–
	*CC*	7	0.04	12	0.05	0.69	0.46	0.26–1.81
	*HSPA1B* (G/A rs1061581)							
	*G*	199	0.56	215	0.52	1	–	–
	*A*	137	0.44	195	0.48	1.3	0.06	0.98–1.76
C	*GG*	41	0.24	48	0.23	1	–	–
	*GA*	117	0.69	119	0.58	1.15	–	–
	**AA**	**10**	**0.05**	**38**	**0.18**	**0.30**	**0.001**	**0.13–0.69**
D	*GG*	41	0.24	48	0.23	1	–	–
	*GA + AA*	127	0.75	157	0.76	0.94	0.82	0.58–1.52
R	*GG + AG*	158	0.94	167	0.81	1	–	–
	***AA***	**10**	**0.05**	**38**	**0.18**	**0.27**	**0.0003**	**0.13–0.57**

*C* codominant, *D* dominant, *R* recessive, *Pc* p value corrected, *IPF* idiopathic pulmonary fibrosis, *OR* odds ratio, *I.C.* confidence interval. Significant associations are highlighted with bold type

**Table 2:** Analysis of multivariate logistic regression of four SNPs of *HSP70* and its association with the IPF for three genetic inheritance models

	B	df	p	OR	95 % C. I.
Codominant model					
*HSPA1* rs1043618					
*GG Vs GC*	−0.72	1	0.16	0.48	0.17–1.36
*GG Vs CC*	−0.28	1	0.59	0.75	0.26–2.12
*HSPA1B* rs1061581					
***GG Vs GA***	**−1.50**	**1**	**0.006**	**0.22**	**0.07–0.65**
***GG Vs AA***	**−1.73**	**1**	**<0.001**	**0.17**	**0.07–0.41**
*HSPA1L* rs2227956					
***AA Vs AG***	**−1.06**	**1**	**0.049**	**0.34**	**0.12**–**0.99**
*HSPA1L* **rs2075800**					
*CC Vs CT*	0.45	1	0.29	1.58	0.67–3.71
***CC Vs TT***	**0.92**	**1**	**0.005**	**2.52**	**1.32**–**4.81**
Dominant model					
*HSPA1L* **rs2227956**					
***AA Vs AG + GG***	**−1.40**	**1**	**0.007**	**0.24**	**0.08**–**0.67**
*HSPA1* **rs1043618**					
***GG Vs GC + CC***	**−0.44**	**1**	**0.039**	**0.63**	**0.41**–**0.97**
*HSPA1B* rs1061581					
*GG Vs GA + AA*	0.17	1	0.63	1.18	0.59–2.39
*HSPA1* rs2075800					
*CC Vs CT + TT*	−0.36	1	0.27	0.69	0.36–1.33
Reccesive model					
*HSPA1* rs1043618					
*GG + GC Vs CC*	−0.35	1	0.47	0.70	0.26–1.85
*HSPA1B* **rs1061581**					
***GG + AG Vs AA***	**−1.78**	**1**	**<0.001**	**0.16**	**0.074**–**0.38**
*HSPA1L* **rs2075800**					
***CC + CT Vs TT***	**0.99**	**1**	**0.001**	**2.70**	**1.46**–**4.98**

*p* p value, *OR* odds ratio, *I.C*. confidence interval. Significant associations are highlighted with bold type

**Fig. 1:**
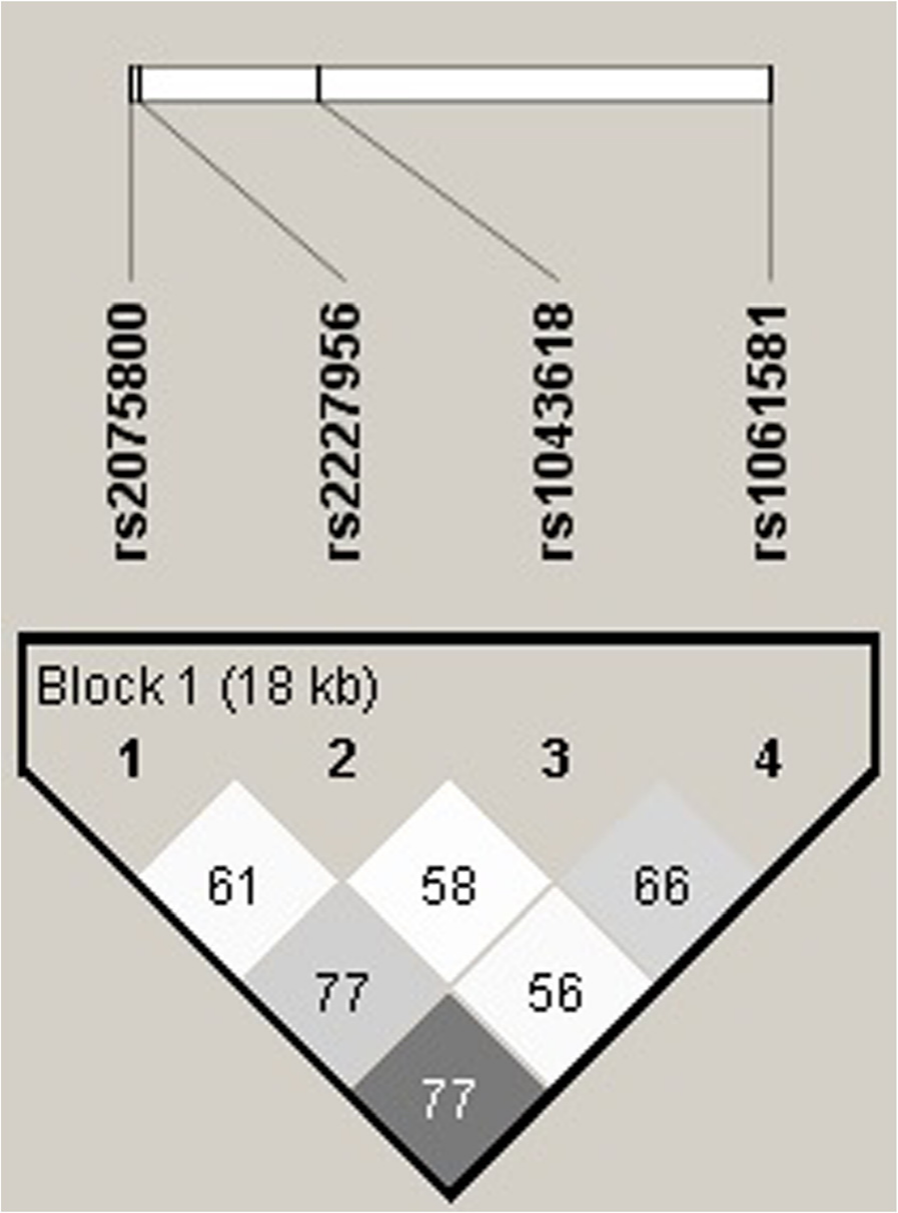
Linkage disequilibrium plot of SNPs in *HSP70* HOM (rs2075800 and rs2227956), *HSPA1A* (rs1043618) and *HSPA1B* (rs1061581), estimated as D´ using Haploview 4.2. The plot was built with data of the control group

Finally, we also performed a haplotype analysis (Table 3) and we found 10 haplotypes [*HSPA1A*- *HSPA1B*- *HSPA1L*] in both studied groups. No significant differences in the distribution of haplotypes between IPF patients and healthy controls were detected. To calculate linkage disequilibrium in the control group the Haploview 4.2 program was used [29]. In Fig. 1, we show the plot of the linkage disequilibrium *HSPA1B* (rs1061581) with *HSP1AL* (rs2075800) and *HSPA1B* (rs1061581) with *HSPA1A* (rs1043618).

**Table 3:** IPF and control haplotype frequencies

Haplotype	Control	IPF
TAGA	0.35	0.30
CACG	0.23	0.13
CAGG	0.22	0.31
CAGA	0.05	0.04
TAGG	0.05	0.09
TACA	0.02	0.02
CGGA	0.02	–
CGGG	0.01	–
CACA	–	0.02
TACG	–	0.03

*IPF* idiopathic pulmonary fibrosis

## Discussion

Hsp70s, together with their Hsp40 co-chaperones, are the most prominent chaperone families that participate in chaperone-assisted proteosomal degradation of misfolded proteins [31]. Previously published functional studies have highlighted the importance of HSP70 to attenuate the abnormal lung remodelling after injury in experimental models [32, 33]. Accordingly, it has been shown that upregulation of HSP70 significantly decreases the inflammatory and fibrotic response in bleomycin-induced pulmonary damage, blocking the production of TGF-β1 [33]. Likewise, there is evidence indicating that gefitinib-induced exacerbation of bleomycin-induced lung fibrosis is mediated by suppression of pulmonary expression of HSP70 [34]. However, studies in human IPF are scarce. Intriguingly, it was found that a subgroup of IPF patients has significantly greater extent of anti-HSP70 humoral and cellular autoreactivities compared with healthy controls. Moreover, abnormal anti-HSP70 humoral autoimmunity was associated to poor outcome. Whether the development of this autoreactivity is related to some *HSP70* polymorphisms is unknown [35]. Interestingly, the autoimmune-associated HLA-B8-DR3 haplotypes seems to include the *HSPA1B* 1267A/G polymorphism [36]. Likewise, it has been reported in the Chinese population that the A allele is more predominant in patients with enterocutaneous fistulas than in healthy controls [37]. In this study, we determined variants of *HSP70* genes in IPF patients and healthy controls from Mexican ancestry. The most striking findings confirmed by a multivariate analysis were that the GA and AA genotypes *of the HSPA1B* (rs1061581) polymorphism were associated with a lower risk of IPF. Also, AG genotype and the AG + GG genotypes (in a dominant model) of the SNP *HSPA1L* (rs2227956) were also associated with a lower risk to develop IPF. On the other hand, the *HSPA1L* (rs2075800) TT genotype was significantly associated with susceptibility to IPF.

The polymorphism rs2227956 is located in the coding region of the *HSPA1L* gene and leads to an amino acid change at position 493 from a non-polar hydrophobic Met to a polar neutral Thr. Amino acid 493 is present in the 18 kDa peptide-binding domain on the beta sheet that forms the floor of the peptide binding groove [38]. The *HSPA1L* polymorphic G allele translates to a Met residue, an hydrophilic amino acid that may affect the interaction of the HSP70 with hydrophobic proteins and consequently impairs its ability to assemble and transport proteins within cells [38, 39]. Intriguingly, a recent meta-analysis study indicated that individuals with *HSPA1B* AG/GG genotype, which seem to protect from IPF, have an increased risk of cancer [40]. Previous studies have highlighted the functional consequences of different HSP-70 gene polymorphisms. The polymorphisms +190 C of *HSPA1A*, *HSPA1B*-179 C>T and *HSPA1B* 1267 A>G have been linked with significant alterations in the mRNA and protein expression [25, 26]. Also an important functional effect at protein level of the polymorphism *HSP70-HOM* +2437 A>G (Met493Thr), affecting the substrate specificity and chaperone activity of HSP-70 HOM, has been described [12, 27]. The possible mechanisms of *HSP70* gene-disease associations in IPF are unclear. In this context, strong evidence has revealed the accumulation of unfolded and misfolded proteins with severe endoplasmic reticulum (ER) stress in alveolar epithelial cells lining areas of fibrosis [41]. It is possible that the functional effect of the *HSPA1B* 1267 A>G polymorphism in the differential expression of *HSPA1B* mRNA [26] might influence the protein expression of pro-fibrotic proteins associated to the pathogenesis of IPF. Moreover, it has been shown that the same cells also display activation of pro-apoptotic pathways [42]. In addition, ER stress may contribute to the pathogenesis of IPF through the induction of epithelial to mesenchymal transition, which may play a role in the expansion of the fibroblast population. Consequently, a transient increase in the expression of heat shock proteins is critical to prevent alveolar epithelial cell death and the shift of epithelial cells to a mesenchymal phenotype. In this perspective, an age-dependent decrease in the ability of different cell types to synthesize HSP70 has been observed, and moreover, some gene variants seem to contribute to this decrease [43].

In our study, the combination of the two genotypes associated with protection was only found in healthy controls and not in IPF patients (*p* = 0.005). In comparison with former data of associated functional polymorphisms [12, 18, 26, 27, 44], it seems that there is no direct relationship among them and those reported herein, since these previous studies show that HSP70 protects from fibrosis development. Strikingly, our results point out that genotypes involved in the decreased induction of these proteins might provide protection against IPF. Moreover, the association between these polymorphisms and IPF protection may be due to LD with other adjacent genes. In this regard, an LD has been reported between the TNF locus and *HSPA1B* [45].

To determine whether the associations described in the three models of inheritance are independent of LD between the SNPs analyzed, a multivariate analysis was performed. As a result we confirmed that these associations are independent of LD. The differences in the association with IPF between the three models of inheritance may indicate that the segregation of one or two genotypes of protection or risk act independently, either as protective or risk IPF factors, respectively. The present study has some limitations and replications in other populations are needed to verify these findings, since polymorphisms suggesting fibrosis protection are relatively rare. Notwithstanding, we were able to confirm *HSP70* polymorphisms association with the protection to IPF with a statistical power greater that 80 %, even with the stringently defined number of IPF cases studied. Another limitation was the imbalanced distribution of males and females among IPF and control group. In this regard, a possible bias of the genetic association analysis may be due to the gender disparities. However, in previous genetic-association studies of MHC genes, no marked differences in the MHC genes frequencies have been detected between males and females from Mexican ancestry [6]. Furthermore, it is well know that IPF is more frequent in Mexican males and due to the age of onset of the disease, it is more frequent to have healthy females than males.

Finally, we compared the frequencies of *HSP70* SNPs genotypes between Mexicans and other ethnic groups (Additional file 1: Table S1). In this analysis we found that the homozygous TT genotype of the polymorphism rs2075800 of the *HSPA1L* gene is the most common genotype, whereas CC genotype is the least frequent genotype in Italian Caucasians [46] and Croatian [47] subjects. We also observed that in Asian populations including Chinese [48] and Taiwanese [49] as well as in Mexicans, the CC genotype is frequent and the genotype TT is uncommon. The genotypic frequencies *HSPA1L* with rs2227956 are similar in all population groups. Likewise, for the *HSPA1A* rs1043618 gene, the GG genotype is more frequent in Chinese, Taiwanese, Polish [50, 51] and Mexicans; in contrast, the most common in Italians is heterozygous CG, and the least frequent in all populations, is the homozygous CC.

## Conclusions

In summary, the present study suggests that genetic variation in the *HSPA1L* and *HSPA1B* genes may influence the susceptibility of developing IPF in our population. Additional investigations with other populations are needed to confirm the significance of our findings and the functionality properties of these polymorphisms.

## Additional file

Additional file 1: Table S1. Genotype frequencies of HSPA1L, HSPA1A and HSPA1B in different populations. (DOC 55 kb)

## Abbreviations

IPF: Idiopathic pulmonary fibrosis
MHC: Major histocompatibility complex
HSP70: Heat shock proteins
SNP: Single nucleotide polymorphism
ECM: Extracellular matrix
TOLLIP: Toll-interacting protein
MICA: MHC class I chain-related gene A
ER: Endoplasmic reticulum
UPR: Unfolded protein response
DNA: Desoxiribonucleic acid
HWE: Hardy–Weinberg equilibrium
OR: Odds ratio
TGF-β1: Transforming growth factor beta 1
HLA: Human leukocyte antigen
LPS: Lipopolysaccharide
