# Supplementary material for: Analysis of heat shock protein 70 gene polymorphisms Mexican patients with idiopathic pulmonary fibrosis
Source: BMC Pulm Med. 2015 Oct 24;15:129. doi: 10.1186/s12890-015-0127-7 (PMC4619986; doi:10.1186/s12890-015-0127-7)
Supplement: Additional file 1: Table S1. — Genotype frequencies of HSPA1L, HSPA1A and HSPA1B in different populations. (DOC 55 kb) [file 12890_2015_127_MOESM1_ESM.doc]

**Additional file 1: Table S1.** Genotype frequencies of HSPA1L, HSPA1A and HSPA1B in different populations.

| HSPA1L C:T rs2075800 | | | | | | | | | | | | | | | | | |
| --- | --- | --- | --- | --- | --- | --- | --- | --- | --- | --- | --- | --- | --- | --- | --- | --- | --- |
| Reference | | (our study) | | | | Guo H | | | Maggioli | | | Chien | | | Matokano | | |
| Population | | Mexican | | | | Chinese | | | Italian | | | Taiwanese | | | Croatian | | |
|  | | n | gf | | | n | gf | | n | gf | | n | gf | | n | gf | |
| Genotype | | 205 | | | | 1152 | | | 388 | | | 178 | | | 95 | | |
| CC | | 59 | 0.28 | | | 440 | 0.38 | | 10 | 0.03 | | 54 | 0.30 | | 1 | 0.01 | |
| TC | | 112 | 0.54 | | | 550 | 0.47 | | 56 | 0.14 | | 99 | 0.56 | | 29 | 0.30 | |
| TT | | 34 | 0.16 | | | 162 | 0.14 | | 322 | 0.83 | | 25 | 0.14 | | 65 | 0.68 | |
| HSPA1L A:G rs2227956 | | | | | | | | | | | | | | | | | |
| Reference | | (our study) | | | | Guo H | | | Kowalcz | | | [Zhang H](http://www.ncbi.nlm.nih.gov/pubmed?term=Zhang H%5BAuthor%5D&cauthor=true&cauthor_uid=21471543) | | |  |  | |
| Population | | Mexican | | | | Chinese | | | Polish | | | Chinese Han | | |  |  | |
|  | | n | | gf | | n | gf | | n | gf | | n | gf | |  |  | |
| Genotype | | 205 | | | | 1152 | | | 243 | | | 294 | | |  | | |
| AA | | 184 | | 0.89 | | 674 | 0.58 | | 174 | 0.71 | | 195 | 0.66 | |  |  | |
| AG | | 21 | | 0.10 | | 412 | 0.35 | | 64 | 0.26 | | 92 | 0.31 | |  |  | |
| GG | | 0 | | 0.00 | | 66 | 0.05 | | 5 | 0.02 | | 7 | 0.02 | |  |  | |
| HSPA1A G:C rs1043618 | | | | | | | | | | | | | | | | | |
| Reference | | (our study) | | | | Guo H | | | Kowalcz | | | Maggioli | | | Chien | | |
| Population | | Mexican | | | | Chinese | | | Polish | | | Italian | | | Taiwanese | | |
|  | | n | | gf | | n | gf | | n | gf | | n | gf | | n | gf | |
| Genotype | | 205 | | | | 1152 | | | 243 | | | 427 | | | 178 | | |
| GG | | 102 | | 0.49 | | 564 | 0.49 | | 135 | 0.55 | | 151 | 0.35 | | 82 | 0.46 | |
| CG | | 91 | | 0.44 | | 486 | 0.42 | | 94 | 0.38 | | 242 | 0.57 | | 78 | 0.44 | |
| CC | | 12 | | 0.05 | | 102 | 0.08 | | 14 | 0.05 | | 34 | 0.08 | | 18 | 0.10 | |
| HSPA1B G:A rs1061581 | | | | | | | | | | | | | | | | | |
| Reference | | (our study) | | | | Kowalc | | | Maggioli | | | Zhang Y | | | Matokano | | |
| Population | Mexican | | | | Polish | | | Italy | | | Chinese | | | Croatian | | |  |
|  | | n | | gf | | n | gf | | n | gf | | n | gf | | n | gf | |
| Genotype | | 205 | | | | 243 | | | 427 | | | 386 | | | 95 | | |
| GG | | 48 | | 0.23 | | 36 | 0.14 | | 42 | 0.10 | | 103 | 0.26 | | 5 | 0.05 | |
| GA | | 119 | | 0.58 | | 107 | 0.44 | | 183 | 0.43 | | 174 | 0.45 | | 44 | 0.46 | |
| AA | | 38 | | 0.18 | | 100 | 0.41 | | 202 | 0.47 | | 109 | 0.28 | | 46 | 0.48 | |

Data correspond to genotype frequencies from healthy controls.

References of Table 4.

Guo H, et al (2011). *Cancer Research* 71 (24): 7576–86; Maggioli E, et al (2014). *International Journal of Immunogenetics* 41 (1): 44–53; Chien CY, et al (2012). *Audiology & Neuro-Otology* 17 (6): 381–85; Matokanović M, et al (2012). *Clinical Biochemistry* 45 (10-11): 770–74; Kowalczyk M, et al (2014). Cell Stress & Chaperones 19 (2): 205–15; Zhang H, et al (2011). In Vivo. 25(2): 251–57; Zhang Y, et al (2013). Cell Stress & Chaperones, 18(6), 703-9 (43).
